# Ohio State Dental Society - Address of Dr. Geo. W. Keely, Retiring President

**Published:** 1872-01

**Authors:** 


### OHIO STATE DENTAL SOCIETY—ADDRESS OF DR. GEO. W. KEELY, RETIRING PRESIDENT.

*Gentlemen of the Ohio State Dental Society:*

At this, our sixth annual meeting, our devout gratitude is due to the Supreme Ruler of the Universe for having so graciously bestowed upon us, through the vicissitudes of the past year, the inestimable blessings of health and a general prosperity. We should surely fail in performing our whole duty if we suffered these bounties of Providence to be bestowed in vain, and not receive them gratefully and apply them industriously for the advancement of our general interest.

During the past year there has been a marked improvement in the steady advance made in the dental profession throughout the State—particularly in the more ennobling feature of saving the natural teeth. May we not justly claim that the influence of this Society had much to do in bringing about this commendable emulation to excel in this special department?

I feel that I can not let this occasion pass without congratulating you upon the *high toned* position taken by this Society—being in advance of any other.

At its organization a committee of most experienced members was appointed, whose duty it was to examine every applicant for membership, thoroughly, as to his professional qualifications, and to investigate, as far as possible, his moral and professional standing in the community where he resided.

Such great caution has been observed in this direction, that, up to the present time, no member has been arraigned for violating the "Code of Ethics," or for unprofessional conduct of any description.

It is true (and we regret its mention), that a few, who were with us for a time, have been dropped from the roll of membership for continued absence and non-payment of dues. This is a great source of regret to us; for our object is mutual improvement and the regeneration of the profession in our State.

These *absent ones* will never have a realizing sense of their great loss; but far *greater* is the misfortune attending the confiding patients they serve.

We also mutually agreed not to take a student, unless he possessed a good English education and good moral character, and for a pupilage not less than two years, and pledging him to take a regular course of lectures, at some reputable dental college, at the close of his service.

This was a stride forward, taken, perhaps, by no other society in existence. A few men have adhered to this idea for years.

But when a society, with the controlling influence of ours, binds its members to such a principle, its benign influence will be felt and a rapid advance made. This society was the first to institute an organized effort in petitioning its State Legislature to pass a law regulating the practice of dentistry.

The accomplishment of this much coveted object required time, patience and untiring perseverance on the part of those who knew no such word as fail.

We have in this law all that the most sanguine should reasonably demand. The compliance with its provisions, thus far, has not come up to the expectations of its friends, although many have gone before the State Board of Examiners and qualified themselves to practice.

And now, as the time is near at hand when all who are practicing dentists in Ohio will be required to have a certificate, as proof of being law-abiding men, it is the imperative duty of this Society to take some definite action in regard to the enforcement of its provisions.

At our last meeting it was unanimously resolved, "that no one shall be eligible to membership in this Society, unless he have a diploma from some reputable dental college or a certificate from the State Board of Examiners."

This action was certainly eminently proper and right, and a duty this Society owed to itself and its reputation.

The Legislature imposed upon us the responsibility of the appointment of a Board of Examiners, and the time had then come when this step should be taken.

Gentlemen, the fondest wish of my heart is that every dentist in our whole country should be fully qualified to practice, and that every one in our great State should become active and live working members of this body.

God grant that the time may soon come, when an intelligent people will demand of every operator all we are endeavoring to accomplish, and when no more banners will be seen "hanging on the outer walls," disgracing an honorable profession.

We organized for the vigorous fight had with the "Boston Monopoly," and within the coming year its continued extortionate and unjust demands should be legally tested, and success will doubtless attend the effort.

I have nothing further to say on this subject, in addition to what was so ably set forth last year by my predecessor.

I would earnestly recommend that this Society take measures to raise a fund to endow professorships in the "Ohio College of Dental Surgery," and for the purpose of erecting a new and more commodious edifice in some more eligible location at Cincinnati.

The College is owned and controlled by an association of stockholders, nearly all of whom are members of the dental profession.

Every one should feel it his duty and privilege to contribute his mite for the benefit of this institution, which has been doing such a noble work during the last quarter of a century.

I would also suggest rescinding the by-law requiring the appointment of a committee to nominate candidates for the various offices for each ensuing year.

Under this rule it is the general understanding that one of the two candidates presented for each office must be elected, which has been the result up to the present time.

I would have no member of this body thus trammeled, but let the question be an open one, and every one free to vote for whom he prefers; with the distinct understanding that he who "works the wires" for his own elevation will certainly insure his defeat.

As the ante-rooms attached to this hall are for the convenience of the various committees, it is desirable they be reserved for this purpose, and the dental depots be located at Major Blount's Hotel.

Your Executive Committee have furnished you with subjects for discussion which claim your careful attention; and this, with the regular order of business and the various miscellaneous matters incident to our meetings, furnish you a great amount of labor to perform.

Attend to one thing at a time. I beg of you not to burthen the Chair with unnecessary motions and amendments.

Concentrate your thoughts, and speak directly to the subject under discussion. Give us the substance—and if any of you have "Bunkum" speeches reserve them for occasions less important.

Be courteous in debate, ever remembering how easy it is to be polite and gentlemanly.

Thanking you for your patient and kind attention, I bid you welcome to your duties—welcome to your labors, and thrice welcome to your pleasures in social intercourse.

